# Pre-Evaluated Safe Human iPSC-Derived Neural Stem Cells Promote Functional Recovery after Spinal Cord Injury in Common Marmoset without Tumorigenicity

**DOI:** 10.1371/journal.pone.0052787

**Published:** 2012-12-27

**Authors:** Yoshiomi Kobayashi, Yohei Okada, Go Itakura, Hiroki Iwai, Soraya Nishimura, Akimasa Yasuda, Satoshi Nori, Keigo Hikishima, Tsunehiko Konomi, Kanehiro Fujiyoshi, Osahiko Tsuji, Yoshiaki Toyama, Shinya Yamanaka, Masaya Nakamura, Hideyuki Okano

**Affiliations:** 1 Department of Orthopedic Surgery, School of Medicine, Keio University, Tokyo, Japan; 2 Department of Physiology, School of Medicine, Keio University, Tokyo, Japan; 3 Central Institute for Experimental Animals, Kanagawa, Japan; 4 Department of Orthopedic Surgery, National Hospital Organization Murayama Medical Center, Tokyo, Japan; 5 Department of Orthopedic Surgery, Saitama Social Insurance Hospital, Saitama, Japan; 6 Center for iPS Cell Research and Application (CiRA), Kyoto, Japan; Chiba University Center for Forensic Mental Health, Japan

## Abstract

Murine and human iPSC-NS/PCs (induced pluripotent stem cell-derived neural stem/progenitor cells) promote functional recovery following transplantation into the injured spinal cord in rodents. However, for clinical applicability, it is critical to obtain proof of the concept regarding the efficacy of grafted human iPSC-NS/PCs (hiPSC-NS/PCs) for the repair of spinal cord injury (SCI) in a non-human primate model. This study used a pre-evaluated “safe” hiPSC-NS/PC clone and an adult common marmoset (*Callithrix jacchus*) model of contusive SCI. SCI was induced at the fifth cervical level (C5), followed by transplantation of hiPSC-NS/PCs at 9 days after injury. Behavioral analyses were performed from the time of the initial injury until 12 weeks after SCI. Grafted hiPSC-NS/PCs survived and differentiated into all three neural lineages. Furthermore, transplantation of hiPSC-NS/PCs enhanced axonal sparing/regrowth and angiogenesis, and prevented the demyelination after SCI compared with that in vehicle control animals. Notably, no tumor formation occurred for at least 12 weeks after transplantation. Quantitative RT-PCR showed that mRNA expression levels of human neurotrophic factors were significantly higher in cultured hiPSC-NS/PCs than in human dermal fibroblasts (hDFs). Finally, behavioral tests showed that hiPSC-NS/PCs promoted functional recovery after SCI in the common marmoset. Taken together, these results indicate that pre-evaluated safe hiPSC-NS/PCs are a potential source of cells for the treatment of SCI in the clinic.

## Introduction

Stem cell-based approaches, such as the transplantation of neural stem/progenitor cells (NS/PCs) into sites of damage, are promising therapies for the alleviation of various central nervous system disorders [1], [2], [3]. Previously, we demonstrated the effectiveness of NS/PC transplantation for the treating spinal cord injury (SCI) in rodents [4] and non-human primates [5]. Considering the differences in the anatomy and functions of the spinal cord between rodents and primates, it was crucial to obtain the proof of concept on the effectiveness of human fetal NS/PCs transplantation for SCI in non-human primates. Nevertheless, we are currently unable to progress toward testing the efficacy of NS/PCs in clinical trials involving humans because of ethical issues surrounding the use of fetus-derived cells. To overcome this hurdle, we have instead focused our efforts on induced pluripotent stem cells (iPSCs) [6], [7] and have demonstrated the utility of “safe” NS/PC clones derived from murine and human iPSCs (iPSC-NS/PCs) in a rodent model of SCI [8], [9].

The present pre-clinical study investigated the therapeutic potential of transplanting pre-evaluated NS/PCs (hiPS-NS/PCs) derived from the safe human iPSC clone, 201B7, into the injured spinal cord of a non-human primate, the adult common marmoset (*Callithrix jacchus*). Grafted hiPSC-NS/PCs survived and differentiated into all three neural lineages (i.e., neurons, astrocytes, and oligodendrocytes), with no evidence of tumor formation. Furthermore, hiPSC-NS/PCs prevented demyelination at the lesion epicenter and enhanced axonal sparing/regrowth and angiogenesis, thereby promoting functional recovery after SCI. These findings suggest that pre-evaluated safe hiPSC-NS/PCs are a promising source of cells for clinical intervention following SCI.

## Materials and Methods

### Animals

All interventions and animal care procedures were performed in accordance with the Laboratory Animal Welfare Act, the Guide for the Care and Use of Laboratory Animals (National Institutes of Health, USA), the Guidelines and Policies for Animal Surgery provided by the Animal Study Committee of the Central Institute for Experimental Animals and Keio University and the guidelines outlined by Weatherall Report,and were approved by the Animal Study Committee of Keio University (IRB approval number 09091-8). Adult female common marmosets (>2 y old) were purchased from a CLEA Japan (Tokyo, Japan). The animals were kept at 26°C with 65% humidity and illumination for 12 h/d. All the animals had free access to food and water in the cage.

### Cell culture, neural induction, and lentivirus transduction

Cell culture and neural induction of hiPSCs (clone 201B7) were performed as described previously [10], with slight modifications. Briefly, hiPSCs (201B7) grown on gelatin-coated (0.1%) tissue culture dishes were maintained in standard ES cell medium and used for EB formation as described previously [10], [11]. EBs were then enzymatically dissociated into single cells and cultured in suspension in serum-free media hormone mix (MHM) media for 10–14 days to allow the formation of neurospheres. Neurospheres were dissociated into single cells and then cultured in the same manner for passage. To assay the degree of differentiation, neurospheres were plated on poly-L-ornithine/fibronectin-coated cover glasses and allowed to differentiate for 10–12 days as previously reported [12]. The lentivirus was prepared and transduced into neurospheres according to previously described methods [9]. Briefly, the concentrated virus particles were added to the culture medium to infect the hiPSC-NS/PCs. 12 days later, human iPSC-derived primary neurospheres were dissociated and infected with lentivirus-expressing Venus fluorescent protein under the control of the elongation factor (EF) promoter (pCSII-EF-Venus). This vector enabled the detection of grafted cells as fluorescent Venus signals using anti-green fluorescent protein (GFP) antibody in fixed spinal-cord sections, since the Venus was originally modified from GFP [13]. This virus was obtained as previously reported [9]. “The titer of the concentrated virus was 1×10^8^–2×10^8^ transducing units per milliliter (TU/ml) when assayed using 293T cells, and infectivity was determined by fluorescence expression, which was analyzed using a FACSCalibur flow cytometer (Becton-Dickinson, Franklin, Lakes, NJ). The primary neurospheres were then passaged into secondary and tertiary neurospheres and used for transplantation.

### Contusive SCI in common marmosets

Adult female common marmosets (*C. jacchus*; CLEA Japan Inc., Tokyo, Japan) were anesthetized with an intramuscular injection of ketamine (50 mg/kg; Sankyo Co., Ltd., Tokyo, Japan) and xylazine (5 mg/kg; Bayer AG, Leverkusen, Germany), followed by inhalation of isoflurane (Fluren; Abbott Japan Co., Ltd., Tokyo, Japan). A moderate contusive SCI was induced in 10 marmosets using a modified NYU (New York University) weight-drop device, as previously reported [5], [14], [15]. Briefly, a 17 g weight (3.5 mm in diameter) was dropped from a height of 50 mm onto the exposed dura mater at the C5 level. During the surgical procedures, the physiological condition of the experimental animals was continuously monitored by electrocardiogram, transcutaneous pulse oximetry (which estimates O_2_ saturation), and both skin and rectal temperature. Nine days after injury, partially dissociated hiPSC-NS/PCs (approximately 1×10^6^ cells/5 µl in cell culture medium without added growth factors) were injected into the lesion epicenter using a glass pipette fitted to a 25-ll Hamilton syringe and a microstereotaxic injection system (David Kopf Instruments, Tujunga, CA). An equal volume of phosphate buffered saline (PBS) was injected into the lesion site for vehicle control common marmosets.

The animals were placed in a temperature-controlled chamber until thermoregulation was reestablished. Manual bladder expression was carried out twice a day until voiding reflexes were reestablished. Paralyzed animals were given adequate amounts of food and water until they recovered their ability to ingest food and water without assistance. Thereafter, they had free access to food and water in the cage.

All animals received daily ampicillin (100 mg/kg; Meiji Seika Kaisha, Ltd., Tokyo, Japan) for 1 week after cell transplantation and subcutaneous cyclosporine injections (10 mg/kg; Novartis, Basel, Switzerland) until they were sacrificed for analysis. At 12 weeks after cell engraftment, animals were anesthetized by intramuscular administration of a mixture of ketamine (50 mg/kg; Sankyo Lifetech Co., Ltd., Tokyo, Japan), xylazine (5 mg/kg; Bayer, Leverkusen, Germany), and atropine (0.02–0.05 mg/kg; Mitsubishi Tanabe Pharma Corporation, Osaka, Japan) prior to magnetic resonance imaging (MRI) scanning. Once anesthetized, the animals were given a mixture of oxygen and 1.5–2.0% isoflurane (Abbott Japan, Tokyo, Japan) via a tracheal intubation tube at a constant rate using an artificial respirator (SN-480-7; Shinano, Tokyo, Japan). The animals were placed on their side in a specially designed body holder (Qualita Ltd., Saitama, Japan). During the scan, the physiological condition of the experimental animals was continuously monitored by electrocardiogram, transcutaneous pulse oximetry and both skin and rectal temperature. Animals were given replacement fluids both before and after the scan. After MRI scanning, animals were euthanized in a humane manner via deep anesthesia (intravenous sodium pentobarbital, 100 mg/kg) for histological analysis. All procedures were approved by the Ethics Committee of Keio University (Tokyo, Japan) and were in accordance with the Guide for the Care and Use of Laboratory Animals (National Institutes of Health, Bethesda, MD).

### Open field test

All behavioral tests were performed from the time of the initial injury up until 12 weeks after SCI. The original open field rating scale was used to evaluate open field locomotion, as described previously [16]. Briefly, all marmosets were individually tested for 5 min on the floor and then in the cage to obtain the overall score (maximum 30 points). During the open field test, marmosets were encouraged by slight tapping if they remained stationary for longer than 15 seconds. In cases of borderline locomotor performance or disagreement between examiners, scores indicating the greater deficit were assigned. The test was performed three times a week up until 4 weeks after SCI, and once a week thereafter. All lower limb and trunk movements except those that were obviously part of a reflex (i.e., spastic extensive bilateral flexion or extension of the hip, knee, and ankle joint) were assessed to obtain the lower limb and trunk score (maximum 10 points). The movements of each bilateral upper limb were separately assessed to obtain the upper limb score, which was defined as the mean of the two independent upper limb scores (maximum 20 points).

### Bar grip test

The motor function of the upper extremities was evaluated using a bar grip strength test, which examines the animal's gripping reflex (the motion undertaken when attempting to grasp an object placed before the animal). The test was performed three times per day, and the maximal daily grip strength was expressed as a percentage of the pre-injury grip strength before SCI [14].

### Cage climbing test

The cage climbing test was developed to evaluate the coordination of the fore and hind limbs. A seven-point scale (from 0 to 6) was used to evaluate the motor coordination of each common marmoset over a period of 3 min. The evaluations were conducted twice a day from immediately before the injury up until 12 weeks after the injury.

### Magnetic resonance imaging

From the viewpoint of the pre-clinical trial, it is critical to evaluate the effect of hiPSC-NS/PCs on the injured spinal cord non-invasively. Therefore, MRI, which enables pathogenic events such as hemorrhage, edema, and cavity formation to be assessed non-invasively [17], [18], was performed at 12 weeks after cell transplantation. MRI was performed by using a 7.0-Tesla superconducting magnet (Bruker Biospin GmbH, Ettlingen, Germany) fitted with a 62 mm inner diameter volume coil under general anesthesia. T2-weighted imaging and Myelin mapping were performed as previously described [19], [20]. Briefly, the non-Gaussian profile for molecular diffusion was directly estimated by performing a Fourier transformation of the data gained from a pulsed gradient spin-echo (PGSE) sequence [21], [22]. An in-house IDL® program was used to perform this analysis. Myelin maps were reconstructed from normalized leptokurtic diffusion data. PGSE sequences were performed using the following parameters: repetition time (TR)/echo time (TE) = 3500 ms/37.8 ms; matrix = 256×192; field of view (FOV) = 40×40 mm^2^; and slice thickness = 1.5 mm. Myelin map-positive myelinated area was quantified using ImageJ software (version 1.29, http://rsbweb.nih.gov/ij/) (n = 5 for each group).

### Histological Analysis

Twelve weeks after cell engraftment, each animal was anesthetized and intracardially perfused with 4% paraformaldehyde (PFA, pH 7.4). Spinal cord tissues were removed, post-fixed in 4% PFA, and immersed overnight in 10% sucrose followed by 30% sucrose. The cord was then embedded in Optimal Cutting Temperature (OCT) compound and sectioned on a cryostat at 20 µm to obtain axial sections. The sections were stained with H-E for general histological examination and with luxol fast blue (LFB) or eriochrome cyanine (EC) to evaluate the extent of the myelination/demyelination after SCI. To quantify the cells of interest in H-E-, LFB-, and EC- stained sections, we obtained images of the stained sections by fluorescence microscopy (BZ-9000; Keyence), manually outlined them, and quantified them using Dynamic cell count BZ-HIC software. The threshold values were maintained at a constant level for all analyses using BZ-HIC. To quantify the area of the cystic cavity, we captured H-E images at the lesion epicenter in axial sections at 100× magnification (n = 5 per group). To quantify the LFB- and EC-positive areas, we automatically captured four regions in axial sections (at the lesion epicenter and 2, 4, and 6 mm rostral and caudal to the epicenter) at 400× magnification (n = 3 per group). To quantify the RT97 positive fibers, the axial sections were scanned at the lesion epicenter, and at 2, 4, and 6 mm rostral and caudal to the epicenter at 400× magnification (n = 5 for each group). To quantify the CaMK-IIα positive fibers, the dorsal corticospinal tract (CST) areas were captured in axial sections of the lesion epicenter at 400× magnification (n = 4 for each group). To quantify the CGRP positive fibers, the dorsal horn areas were captured in axial sections of the 6 mm roatral and caudal to the epicenter at 400× magnification (n = 3 for each group). To quantify the Iba-1-positive areas in axial sections of the lesion epicenter, we captured at the lesion epicenter in axial sections at 10× magnification (n = 3 for each group). For the quantification of PECAM-1 positive blood vessels area, the axial sections were captured at the lesion epicenter at 100× magnification (n = 5 for the transplantation group and 4 for the control group). To quantify the proportion of each cell phenotype *in vivo*, we selected representative mid-sagittal sections and automatically captured five regions within 1 mm rostral and caudal to the lesion epicenter at 200× magnification. Venus-positive engrafted and phenotypic marker-positive cells were counted in each section (n = 4 per group).

Tissue sections were stained with the following primary antibodies: anti-green fluorescent protein (GFP, rabbit IgG, 1∶200; Frontier Institute Co., Ltd., Hokkaido, Japan), anti-GFP (goat IgG, 1∶200; Rockland Immunochemicals, Gilbertsville, PA), anti-β-tubulin isotype III (βIII tubulin, Tuj1, mouse IgG, 1∶1,000; Sigma Chemical Co., St. Louis, MO), anti-HNu (mouse IgG, 1∶500; Chemicon, Temecula, CA), anti-NeuN (mouse IgG, 1∶200; Chemicon), anti-glial fibrillary acidic protein (GFAP) (rabbit IgG, 1∶200; Dako, Carpinteria, CA), anti-GFAP (guinea pig IgG, 1∶200; Advanced ImmunoChemical, Long Beach, CA), anti-GFAP (rat IgG, 1∶200; Invitrogen, Carlsbad, CA), anti-Olig1 (mouse IgG, 1∶200; R&D Systems, Minneapolis, MN), anti-human-specific Nestin protein (rabbit IgG, 1∶200; described previously, refs. [5], [9], [23], [24]), anti-Ki-67 (rabbit IgG, 1∶500; Novocastra), anti-NF-200 (RT97 monoclonal antibody, mouse IgG, 1∶200; Chemicon), anti-PECAM-1 (rat IgG, 1∶50; BD Bioscience Pharmingen, San Diego, CA), anti-CaMK-IIα (CaMK-IIα, mouse IgG, 1∶100; Invitrogen) anti-calcitonin gene-related peptide (CGRP, rabbit IgG, 1∶1,000; Affinity), and anti-Iba-1 (rabbit IgG, 1∶200; Wako)

Images were obtained via fluorescence microscopy with a BZ-9000 fluorescence microscope (Keyence, Woodcliff Lake, NJ) or by confocal laser scanning microscopy with a LSM700 Laser Scanning Microscope (Carl Zeiss Microscopy, GmbH, Munich, Germany).

For immunohistochemistry with RT97, spinal cord tissues were first exposed to 0.3% H_2_O_2_ for 30 min at room temperature to inactivate endogenous peroxidases. The tissues were then reacted with a biotinylated secondary antibody (Jackson ImmunoResearch Laboratories, West Grove, PA). Immunohistochemical signals were enhanced using the Vectastain ABC kit (Vector Labs, Burlingame, CA). Nuclei were stained with Hoechst 33258 (10 µg/mL; Sigma), a DNA-specific fluorochrome.

### RNA Isolation and RT-PCR

Gene expression profiling of hiPSC-NS/PCs and hDFs was performed *in vitro* to evaluate the mRNA expression levels of trophic factors related to axonal regrowth and angiogenesis (NT3, NT4, CNTF, and vascular endothelial growth factor [VEGF]). RNA was isolated from each cell type using TRIzol (Invitrogen) according to the manufacturer's instructions. Total RNA (1 µg) was treated with TURBO DNase (Ambion Life Technologies, Grand Island, NY) and then reverse transcribed using oligo (dT) primers and SuperScript III (Invitrogen). Quantitative mRNA expression levels for the trophic factor-related genes were simultaneously analyzed (n = 2 for each cell type) using quantitative RT-PCR arrays using TaqMan Array Fast (Applied Biosystems) according to the manufacturer's instructions. The following commercially available primers against human DNA sequences (Applied Biosystems, Carlsbad, CA) were useed: GAPDH-Hs99999905_m1, NTF3-Hs00267375_s1, NTF4-Hs01921834_s1, ZFP91-CNTF-Hs00173456_m1, and VEGFA-Hs00900058_m1. The mRNA expression level for each factor was normalized to the level of glyceraldehyde 3-phosphate dehydrogenase (GAPDH) mRNA. The mRNA expression data for the hiPSC-NS/PCs were then calculated as the amount of mRNA for each factor relative to the amount in hDFs.

### Statistical Analysis

All data are reported as the mean ± SEM. For all histological examinations, an unpaired two-tailed Student's t-test was used for single comparisons between the transplantation and vehicle control groups. The results of the open field test, bar grip test, and cage climbing test were analyzed using a Mann-Whitney U-test. In each case, *p<0.05 and **p<0.01 were considered to be statistically significant.

## Results

### Grafted hiPSC-NS/PCs survive and differentiate into all three neural lineages without becoming tumorigenic

**Figure 1:**
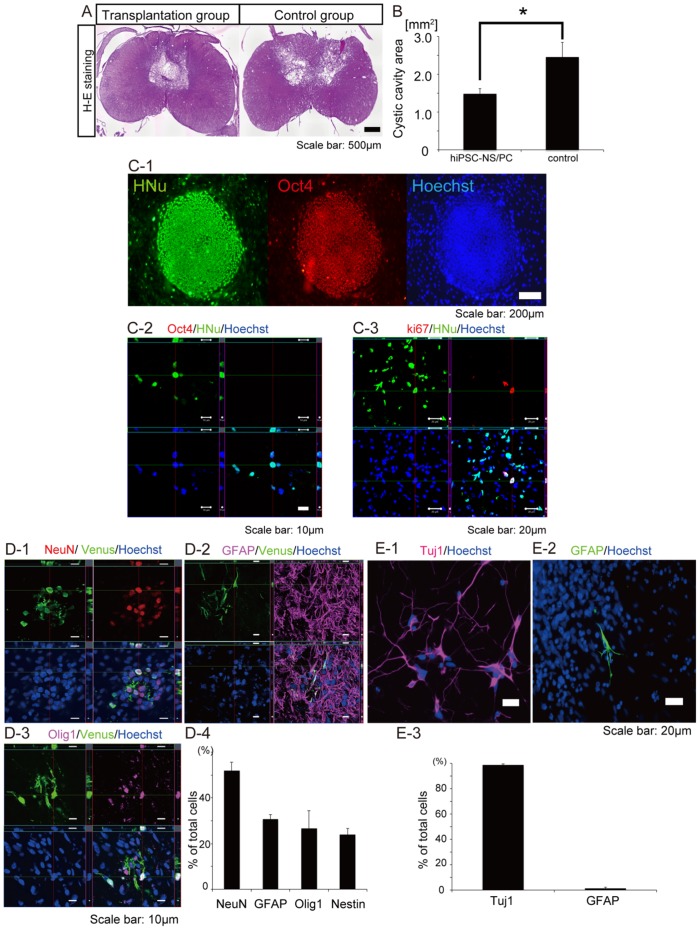
Grafted hiPSC-NS/PCs differentiate into three neural lineages without tumor formation in the injured spinal cord. (**A**) Representative H-E stained images of axial sections at the lesion epicenter at 12 weeks (84 days) after cell transplantation. No tumor formation was observed in the hiPSC-NS/PC-transplanted group during this time. H-E staining showed that cystic cavity formation at the lesion epicenter was prominent in the vehicle control group compared with the transplantation group, whereas there was no clear difference in the transverse area of the injured spinal cord. (**B**) Quantification of the cystic cavity area. Data represent the mean ± the SEM (n = 5 for each group, *p<0.05). (**C**) Immunostaining for Oct4 and Ki-67. (**C-1**) All colonies of undifferentiated iPSCs were positive for Oct4, a marker of undifferentiated ESCs and iPSCs. (**C-2**) Grafted hiPSC- NS/PCs yielded no Oct4-positive following transplantation into the injured spinal cord. (**C-3**) The percentage of HNu-positive cells that were also Ki-67-positive was 0.55±0.08%. (**D**) Differentiation of grafted hiPSC-NS/PCs. (**D-1-3**) Representative images of Venus-positive grafted hiPSC-NS/PCs immunostained with antibodies against NeuN to detect mature neurons, GFAP to detect astrocytes, and Olig1 to detect oligodendrocyte progenitor cells. (**D-4**) Percentages of cell type-specific, marker-positive cells among the Venus-positive grafted hiPSC-NS/PCs at 12 weeks post-engraftment. (**E**) In vitro differentiation of hiPSC-NS/PCs. (**E-1, 2**) Representative images of hiPSC-NS/PCs immunostained with antibodies against βIII-tubulin to detect neurons, and GFAP to detect astrocytes. (**E-3**) Percentages of cell type-specific marker-positive cells among the hiPSC-NS/PCs 10 days after the initiation of cell differentiation in vitro.

A moderate contusive SCI was induced at cervical level 5 (C5) in adult common marmosets as reported previously [14]. Nine days after injury, human iPSC-NS/PCs (1×10^6^ cells/5 µl) were transplanted into the injured spinal cord in the transplantation group. In the vehicle control group, PBS was injected instead of cells. At 12 weeks post-engraftment, hematoxylin-eosin (HE) staining showed that cystic cavity formation was prominent in the vehicle control group compared with the transplantation group (Fig. 1A). A significant difference in the size of the transverse area of the cystic cavity at the lesion epicenter was observed between the two groups (Fig. 1B). Notably, no evidence of tumor formation was observed in any of the animals in the transplantation group at 12 weeks after cell engraftment (Fig. 1A, B).

**Figure 2:**
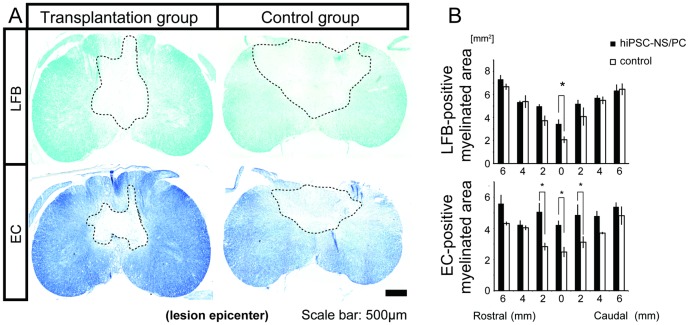
Grafted hiPSC-NS/PCs prevent demyelination after SCI. (**A**) Representative LFB- and EC-stained images of axial sections through the lesion epicenter at 12 weeks post-engraftment. The region enclosed by the dashed line indicates the demylinated area. (**B**) Quantification of the images revealed significant preservation in the size of the myelinated areas at the lesion epicenter in the transplantation group compared with the vehicle control group. Data represent the mean ± SEM (n = 3 per group, *p<0.05).

**Figure 3:**
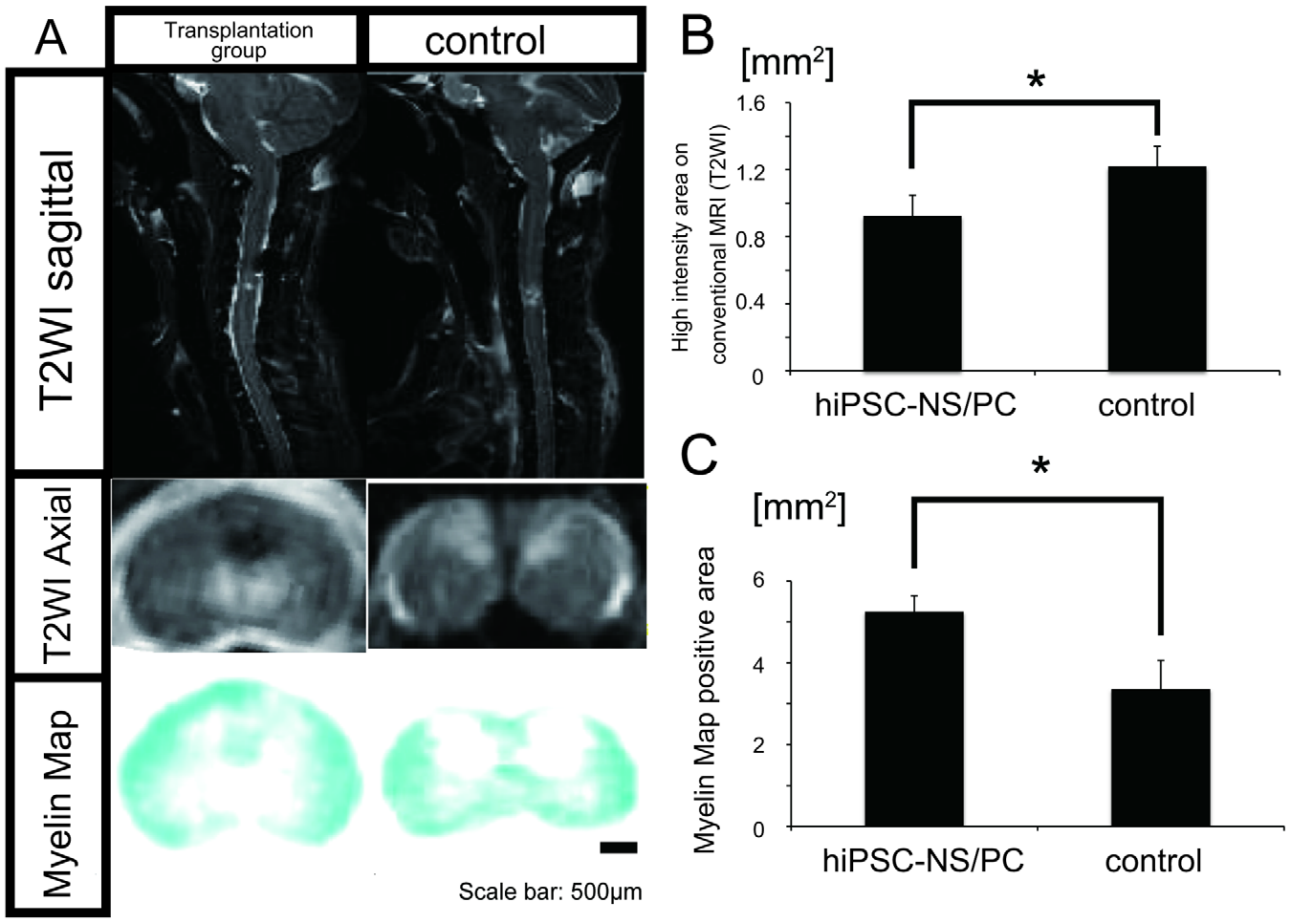
Conventional MRI and construction of a Myelin map. (**A**) MRI and Myelin map of the spinal cord was carried out at 12 weeks after cell engraftment. (**B**) T2WIs obtained by conventional MRI showed that the intramedullary high-signal intensity area at the lesion epicenter was prominent in the vehicle control group compared with the transplantation group. Data represent the mean ± SEM (n = 5 per group, *p<0.05). (**C**) The Myelin map-positive area was increased in the transplantation group relative to that in the control group. Data represent the mean ± SEM (n = 5 per group, *p<0.05).

**Figure 4:**
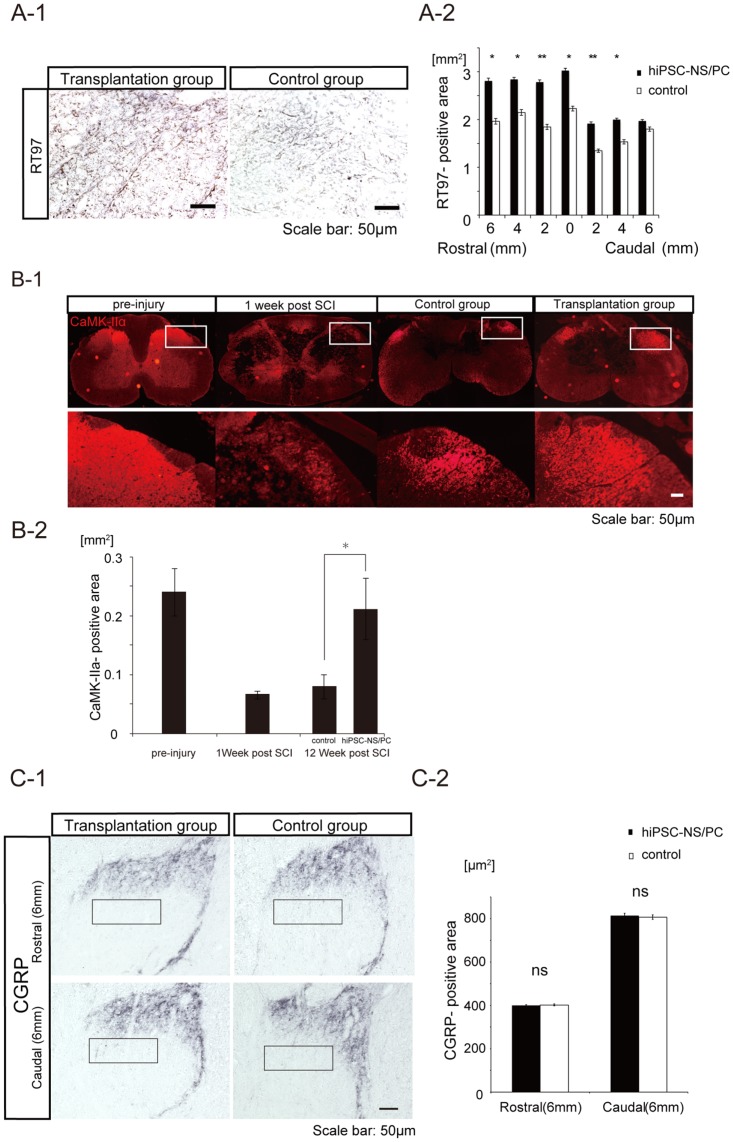
Grafted hiPSC-NS/PCs spare descending motor axons but do not induce abnormal innervations of pain-related CGRP positive afferents after SCI. (**A-1**) Representative images of RT97-positive neurofilament staining in the ventral region of the spinal cord at the lesion epicenter at 12 weeks post-engraftment. (**A-2**) Quantification of RT97-positive areas at the lesion epicenter. The overall RT97-postive area was larger in the transplantation group than in the control group. Data represent the mean ± SEM (n = 5 per group, *p<0.05, **p<0.01). (**B-1**) Representative images of CaMK-IIα-positive descending motor axons in the dorsal corticospinal tract (CST) area of the lesion epicenter. (**B-2**) Quantification of CaMK-IIα-positive areas at the lesion epicenter. The overall CaMK-IIα-positive area was increased in the transplantation group compared with that in the control group at 1 week post SCI. Data represent the mean ± SEM (n = 4 for each group, *p<0.05). (**C-1**) Representative images of CGRP-positive fibers in the dorsal horn 6 mm rostral and caudal to the lesion epicenter. (**C-2**) Quantitative analysis of the CGRP-positive areas in the dorsal horn 6 mm rostral and caudal to the lesion epicenter. There were no significant differences in the size of the CGRP-positive areas in the dorsal horn between the transplantation and control groups. Data represent the mean ± the SEM (n = 3 per group.)

Immunostaining for Oct4, a marker for undifferentiated pluripotent stem cells [25], revealed that undifferentiated iPSCs were all positive for Oct4 (Fig. 1. C-1). On the other hand, no Oct4-positive cells were observed among the grafted human nuclear protein (HNu)-positive hiPSC-NS/PCs (Fig. 1. C-2). We also performed immunostaining for the proliferation marker Ki-67 to determine the level of proliferation of the grafted cells. The percentage of HNu-positive cells that were also Ki-67-positive was 0.55±0.08% (Fig. 1. C-3). Indeed, grafted hiPSC-NS/PCs survived around the lesion epicenter in the host spinal cord and differentiated into mature neuronal nuclei (NeuN)-positive neurons, GFAP-positive astrocytes, and oligodendrocyte transcription factor 1 (Olig1)-positive oligodendrocyte progenitor cells (Fig. 1D-1–D-3). Quantitative analyses revealed that the population of differentiated cells within the host spinal cord consisted of 52.0±3.7% NeuN-positive cells, 30.8±1.9% GFAP-positive cells, 26.5±7.8% Olig1-positive cells, and 23.9±2.8% Nestin-positive cells (Fig. 1D-4). In contrast, hiPSC-NS/PCs primarily differentiated into βIII-tubulin-positive neurons (98.7±0.9%) *in vitro* (Fig. 1E).

### Transplantation of hiPSC-NS/PCs prevents demyelination after SCI

Next, LFB and EC staining were performed to evaluate the effect of hiPSC-NS/PC transplantation on the extent of demyelination/remyelination after SCI. Severe demyelination was evident in the central and posterior parts of the lesion site in both the transplantation group and the vehicle control group, whereas an area of spared myelination was prominent at the rim of the injured spinal cord in the transplantation group (Fig. 2A). Quantification of the myelinated areas in the lesion sites revealed significant differences between the transplantation and control groups at 12 weeks after cell engraftment (Fig. 2B).

Conventional MRI and Myelin map imaging of the spinal cord were conducted at 12 weeks after cell transplantation as described previously [19], [20] (Fig. 3A). T2-weighted images (T2WIs) showed that an intramedullary high-signal intensity area at the lesion site was prominent in the vehicle control group, which was significantly larger than the comparable area in the transplantation group (Fig. 3B). These findings are consistent with the quantitative data discussed above regarding the increased transverse area of the cystic cavity at the lesion epicenter in the vehicle control relative to the transplantation group (Fig. 1B). Furthermore, the Myelin map showed that the overall myelin-positive area at the lesion epicenter was significantly larger in the transplantation group than in the control group (Fig. 3C). These results are also consistent with the quantitative data shown in Fig. 2B regarding the differences in the size of the LFB- and EC-positive transverse myelinated areas between the two groups.

### Grafted hiPSC-NS/PCs enhance axonal sparing/regrowth and angiogenesis

To evaluate the impact of grafted hiPSC-NS/PCs on axonal sparing/regrowth, immunostaining for neurofilament-200 (NF-200, Fig. 4A-1) and α-CaM (Ca^2+^/calmodulin-dependent) kinase II (CaMK-IIα, Fig. 4B-1) was performed. NF-200 (detected by monoclonal antibody RT97) is a marker for mature, large-diameter axons, and CaMK-IIα is a marker for descending motor axons. Immunostaining analysis revealed a greater number of RT97-positive areas at the lesion epicenter as well as at sites adjacent to the lesion in the transplantation group relative to the vehicle control group (Fig. 4A-1). Moreover, quantitative analysis demonstrated that the overall RT97-positive area was larger in the transplantation group than in the control group (Fig. 4A-2). The CaMK-IIα-positive area at the lesion epicenter was also significantly larger in the transplantation group than in the control group at 1 week after SCI. Contusive SCI resulted in a significant decrease in the size of the CaMK-IIα–positive area at the lesion site, which was followed by a slight, but not statistically significant, increase in the control group 12 weeks after SCI. The size of the CaMK-IIα-positive area 12 weeks after SCI was significantly increased in the transplantation group. There was a significant difference in the CaMK-IIα-positive area at the lesion epicenter between the transplantation and the control groups (Fig. 4B-2). Furthermore, We investigated the distribution of calcitonin gene-related peptide (CGRP) fibers, which are involved in peripheral and spinal pain mechanisms [9], [26], [27], [28], to examine the effect of hiPSC-NS/PCs in pain afferents entering the dorsal horn of the spinal cord both above and below the injured spinal segments (Fig. 4C-1). There were no significant differences in the size of the CGRP-positive areas in lamina III between the hiPSC-NS/PCs and the control group (Fig. 4C-2).

**Figure 5:**
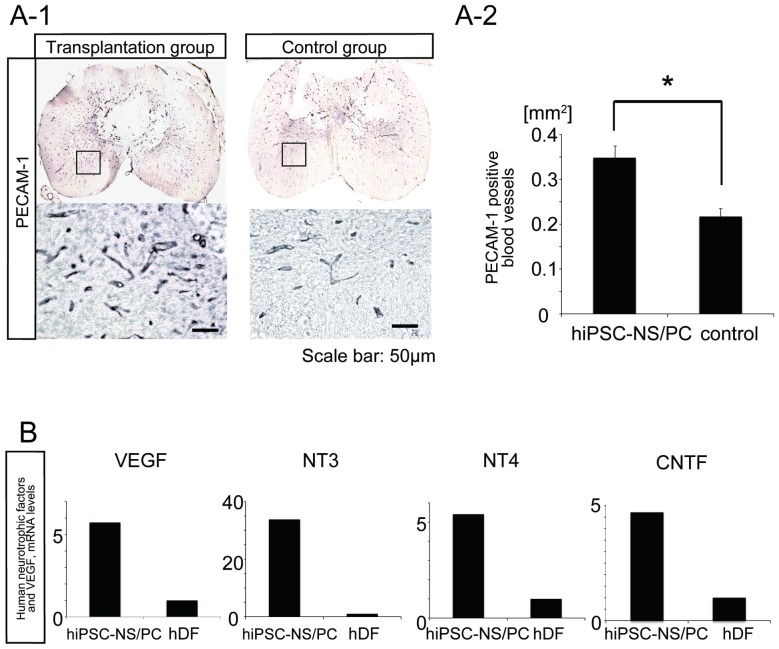
Grafted hiPSC-NS/PCs secrete neurotrophic factors and enhance angiogenesis after SCI. (**A-1**) Representative images of PECAM-1-positive blood vessels at the lesion epicenter. Scale bar: 50 µm. (**A-2**) Quantification of PECAM-1-positive blood vessels. The number of PECAM-1-positive blood vessels per unit area was higher in the transplantation group than in the control group. Data represent the mean ± SEM (n = 5 for the transplantation group and n = 4 for the control group, *p<0.05). (**B**) Quantitative RT-PCR and mRNA expression levels of VEGF and human neurotrophic factors. The expression levels of VEGF, NT3, NT4, and CNTF were all significantly higher in hiPSC-NS/PCs than in hDFs.

Immunohistochemical analysis for platelet endothelial cell adhesion molecule-1 (PECAM-1) was next performed to evaluate the effects of hiPSC-NS/PC transplantation on angiogenesis after SCI (Fig. 5A-1). A significantly larger number of PECAM-1-positive blood vessels per unit area were observed at the lesion epicenter in the transplantation group compared with the vehicle control group (Fig. 5A-2). Taken together, the results of the immunohistochemical study suggest that the transplanted hiPSC-NS/PCs mediated regeneration and/or sparing of axons and generation of new blood vessels in our injury model.

Human dermal fibroblasts (hDFs) are the original cell source of the hiPSC-NS/PCs used in this study. Quantitative RT-PCR of the cultured hiPSC-NS/PCs and hDFs revealed that mRNA expression levels of human neurotrophic factors associated with axonal growth and survival of host neurons (i.e., neurotrophin (NT) 3, NT4, and ciliary neurotrophic factor (CNTF)) [29], [30], [31] were significantly higher in cultured hiPSC-NS/PCs than in cultured hDFs (Fig. 5B). The mRNA expression level for the human pro-angiogenic factor, VEGF [32] was also elevated in cultured hiPSC-NS/PCs. Thus, secretion of NT3, NT4, CNTF, and VEGF may contribute to the regenerative properties of the transplanted stem cells after SCI.

### Microglial activation and glial scar formation at 12 weeks after transplantation

**Figure 6:**
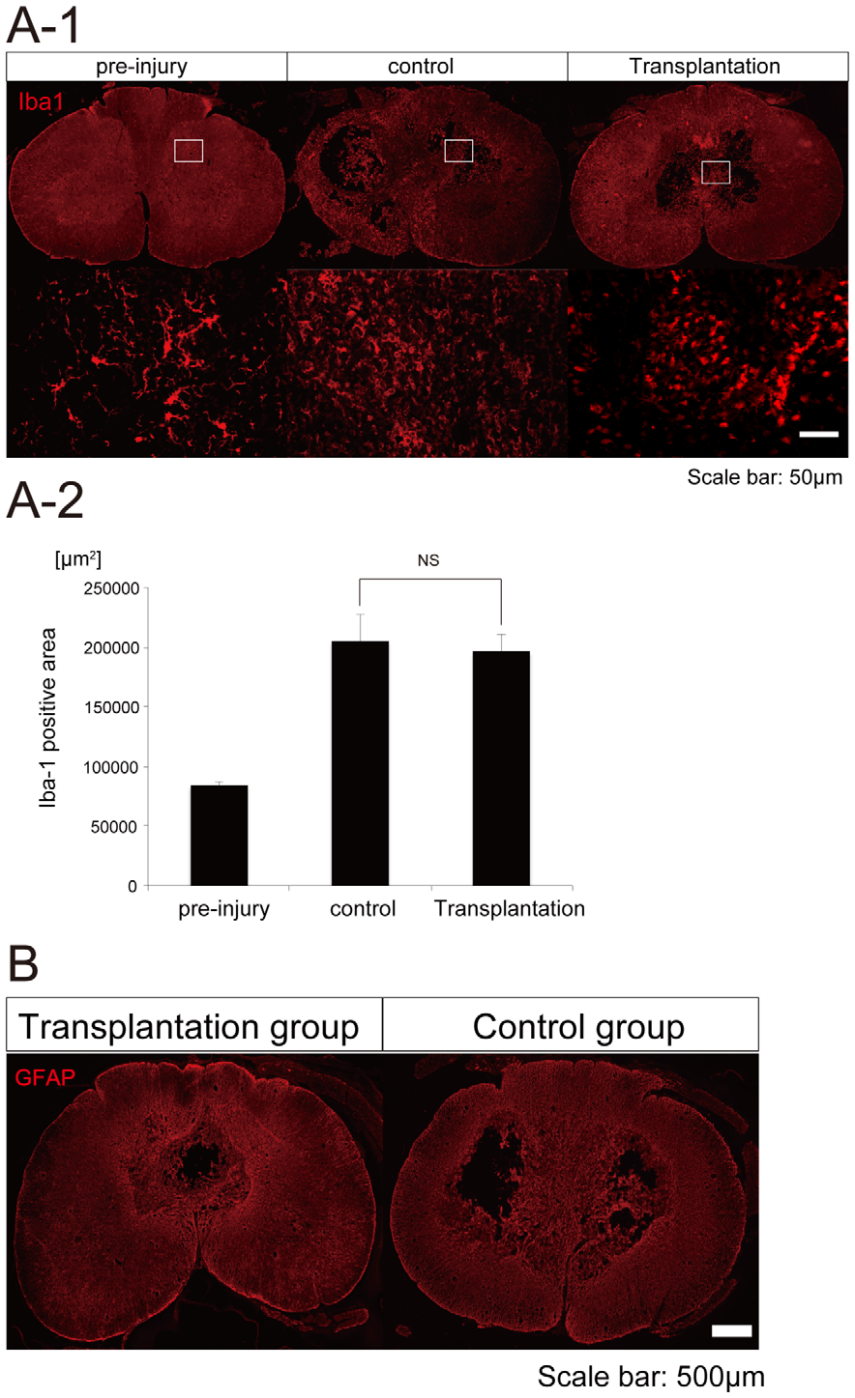
Microglial activation and glial scar formation at 12 weeks after transplantation. (A-1) Representative images of Iba1 staining in the spinal cord at the lesion epicenter at 12 weeks post-engraftment. Iba1-positive microglia showed a more rounded shape in the control and transplantation groups than in the pre-injury sample. (A-2) Quantification of the size of the Iba-1-positive area. The size of the Iba-1-positive area was not significantly different between control and transplantation groups at 12 weeks post-transplantation. (B) Representative GFAP-stained images of axial sections at the lesion epicenter at 12 weeks after cell transplantation. There were no significant differences in the size of GFAP-positive areas between the hiPSC-NS/PCs and the control groups.

We performed Iba1 staining to assess microglial activation and GFAP staining to assess glial scar formation. Iba1-positive microglia showed a more rounded shape in the control and transplantation groups than in the pre-injury sample (Fig. 6A-1). However, we could not detect the difference between control and transplantation groups, nor detect the apparent evidence of immunomodulation by the hiPSC-NS/PCs at this time point (12 weeks after transplantation) (Fig. 6A-2). There were no significant differences in the size of GFAP-positive areas between the hiPSC-NS/PCs and control groups (Fig. 6B).

### Grafted hiPSC-NS/PCs promote functional recovery after SCI

**Figure 7:**
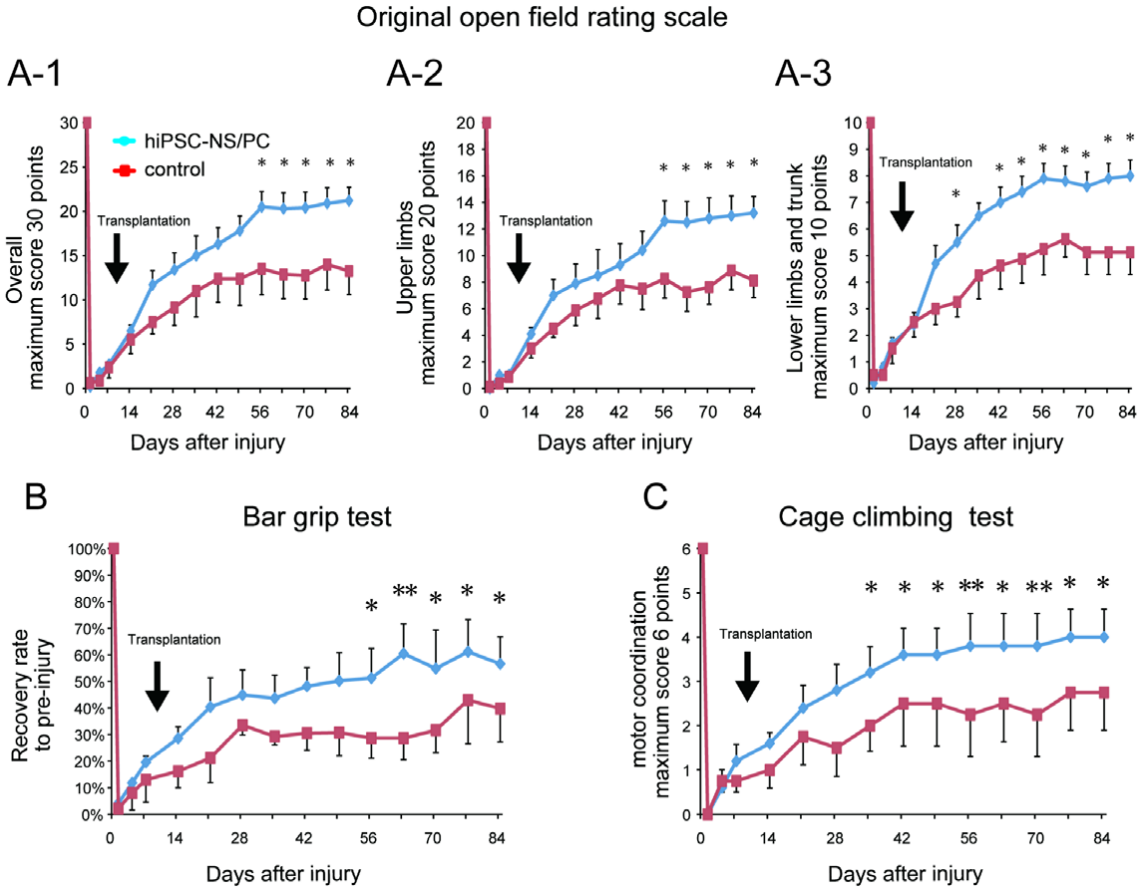
Grafted hiPSC-NS/PCs promote functional recovery after SCI. (**A-1**) Original open field rating scale (maximum 30 points). Immediately after injury, the score decreased to approximately zero for both groups. By 12 weeks after injury, the score had increased to a plateau of ∼21 in the transplantation group and ∼13 in the vehicle control group. Data represent the mean ± SEM (n = 5 animals for the transplantation group and n = 4 for the control group, *p<0.05). (**A-2**, **A-3**) Open field locomotor rating scale for the upper limbs (maximum 20 points) and the lower limbs and trunk (maximum 10 points). Data represent the mean ± SEM (n = 5 for the transplantation group and 4 for the control group, *p<0.05). (**B**) Bar grip test showing the time course of recovery of bar grip strength in the transplantation and the control groups. Each value indicates the percentage of pre-injury bar grip strength. Bar grip strength recovered gradually, reaching a plateau of ∼57% versus ∼40% of the pre-injury value in the transplantation group and the control group, respectively, by 12 weeks after injury. Data represent the mean ± SEM (n = 5 for the transplantation group and n = 4 for the control group, *p<0.05, **p<0.01). (**C**) Cage climbing test. The average test score was zero for both groups immediately after injury. By 12 weeks after injury, the mean score of the animals in the transplantation group had improved to 4, whereas the mean score of the animals in the control group had improved to 2.8. Data represent the mean ± the SEM (n = 5 for the transplantation group and n = 4 for the control group, *p<0.05, **p<0.01).

Contusion SCI at the C5 level caused severe tetraplegia in the adult common marmosets. Tetraplegia was followed by gradual recovery of motor function, as reported previously [14]. Immediately after injury, all of the examined variables in the behavioral tests (the open field test, bar grip test, and cage climbing test) sharply decreased to a mean value of zero in the transplantation group and in the vehicle control group (Fig. 7).

Open field locomotion was evaluated by the original open field rating scale [16]. Briefly, all marmosets were individually tested for 5 min on the floor and then in the cage to obtain the overall score (maximum 30 points). Because this study used a severe central cord injury model rather than a complete injury model, the behavioral scores in the overall original open field rating scale gradually increased from zero and reached a plateau of ∼21 in the transplantation group over the course of the experiment. The corresponding behavioral scores reached a plateau of ∼13 in the vehicle control group (Fig. 7A-1). The results for the vehicle control group are consistent with the clinical course of central cord injury [33], [34]. Significant differences were observed between the two groups in the overall open field behavioral scores at 8 weeks (56 days) following SCI and thereafter (Fig. 7A-1). Significant differences were also observed at 56 days following SCI and thereafter in the open field upper limb scores (Fig. 7A-2), and at 28 days following SCI and thereafter in the open field lower limb and trunk scores (Fig. 7A-3).

The bar grip strength of the forelimbs also increased from a mean value of zero over the course of the experiment and gradually reached a plateau of ∼57% and ∼40% of the pre-injury value in the transplantation group and the control group, respectively, at 12 weeks after injury (Fig. 7B). Similarly, the mean scores of the cage climbing test improved from zero to a value of 4 in the transplantation group and 2.8 in the vehicle control group (Fig. 7C). All of the examined values for the open field test, bar grip test, and cage climbing test were significantly different between the transplantation and vehicle control groups at 8 weeks following SCI and thereafter.

## Discussion

### Rationale for the level of injury, type of injury, and timing of the intervention

It is important that validated experimental approaches in animal models are improved to meet the needs of individuals with SCI and to advance the field of regenerative neuroscience [35]. Because the number of patients with cervical injuries is increasing [36], further work with cervical contusive/compressive SCI models is required in parallel with clinical trials. Therefore, we developed a cervical contusion SCI model in the common marmoset in the present study.

Previous reports show that transient severe inflammation occurs around the injured site during the acute phase of SCI when the levels of many inflammatory cytokines, which have neurotoxic or astrocyte-inducing effects (such as IL-1, IL-6, and TNF), increase and then decline sharply within 24 hr [37]. Therefore, the microenvironment of the injured spinal cord during the acute phase is not suitable for the survival or differentiation of grafted cells. In fact, the transplantation of NS/PCs 9 days after SCI results in mitogenic neurogenesis, whereas this does not occur when the transplantation is performed within a few days of SCI [4], [38]. The chronic phase of SCI is unlikely to be a suitable period for therapeutic transplantation due to the formation of enlarged cysts and the development of glial scarring, which might inhibit axonal regeneration [39], [40]. Based on these studies, we hypothesized that the subacute phase of SCI is the optimal time for NS/PC transplantation.

### Differentiation potential of grafted hiPSC-NS/PCs

In our previous study [9], we evaluated the tumorigenicity of hiPSC-NS/PCs grafted into the injured spinal cords of NOD-Scid mice. Long-term observation after transplantation (3 months) revealed no tumorigenicity of NS/PCs derived from the 201B7 clone. Based on this finding, we consider 201B7-iPS to be a “safe clone”. The present study evaluated the therapeutic potential of these pre-evaluated safe hiPSC-NS/PCs for SCI in adult common marmosets. The grafted hiPSC-NS/PCs differentiated into neurons, astrocytes, and oligodendrocyte progenitor cells *in vivo*, whereas hiPSCs predominantly differentiated into neurons *in vitro*. These findings were consistent with the results of the transplantation of hiPSC-NS/PCs into the injured spinal cord of immunodeficient mice, suggesting that inhibitory factors for neuronal maturation exist within the injured spinal cord. It is also possible that the oligodendrocytes differentiation factors are absent *in vitro*, but present in the injured spinal cord. By contrast, human fetus-derived NS/PCs primarily differentiated into astrocytes following transplantation into the injured spinal cord of common marmosets in an earlier study [5]. Although the microenvironment of the injured spinal cord is critical for the survival and differentiation of grafted NS/PCs [38], these findings suggest that the origin of the NS/PCs plays an important role in their differentiation phenotype following transplantation during the subacute phase of SCI.

### Tumorigenicity of hiPSC-NS/PCs

Because one of the predominant concerns regarding hiPSC-based therapy is the potential tumorigenicity of the grafted cells, animals were observed for tumor formation for up to 12 weeks after cell transplantation. No histological evidence of tumor formation was found during this time in any of the animals; this result was also confirmed by conventional MRI. For example, Oct4 is required for maintaining the pluripotency of embryonic stem cells (ESCs) [25], [41] and for generating iPSCs [6], [7], but is potentially tumorigenic when expressed in grafted hiPSC-NS/PCs [42]. However, histological assessment revealed that the grafted hiPSC-NS/PCs were negative for Oct4. We also examined the amount of proliferation among the grafted cells by Ki-67 labeling. The percentage of HNu-positive cells that were Ki-67-positive cells was 0.55±0.08%. On the other hand, 23.9±2.8% of the grafted hiPSC-NS/PCs remained immature, as determined by their expression of Nestin within the host spinal cord at 12 weeks (84 days) after transplantation; the percentage of Nestin-positive hiPSC-NS/PCs was similar to that of human fetus-derived NSCs (25.2±2.9%) at 56 days after transplantation into the injured spinal cord of common marmosets [5]. Interestingly, when NS/PCs derived from the same hiPSC clone were grafted into the injured spinal cord of immunodeficient mice in our previous study [9], lower percentages of Nestin-positive hiPSC-NS/PCs were observed (10.7±2.2% at 56 days and 7.5±1.0% at 112 days after transplantation). Thus, differentiation of transplanted cells might be influenced not only by the origin of the NS/PCs, but also by species-specific differences in the microenvironment afforded by the host spinal cord. Strictly, we cannot exclude the risk of tumorigenesis derived from these Nestin-positive undifferentiated neural progenitor cells after 16 weeks post-transplantation. Thus, considering the fail safe system of future clinical trial, we should establish the selective ablation of the grafted-cell using such as the herpes simplex thymidine kinase (HSV-tk) and “suicide” gene system [43], [44], if tumor formation occurred after human iPS-NS/PC transplantation.

### Mechanism of functional recovery after hiPSC-NS/PCs transplantation

Stem cell-based therapies for SCI could hypothetically affect histological and/or functional outcomes through a number of beneficial mechanisms, including axonal regeneration, local-circuitry reconstruction, neurotrophic effects, angiogenesis and immunomodulation, all of which have been reported to regenerate the damaged spinal cord in previous studies using rodent ESCs/iPSCs [8], [9], [45]. Compared with rodents, non-human primates such as common marmosets have distinct differences in terms of anatomy, functional neural pathways in the spinal cord, and immune responses. Although it is difficult to evaluate the functional recovery of the forelimbs in the rodents, this is possible in the SCI model of the non-human primates. Furthermore, to validate and predict the therapeutic effects of interventions used as human treatments, contusive injury is considered to be the most relevant to human SCI. Thus, the present study using common marmosets could be important for the pre-clinical evaluation of human iPSC therapy for human SCI.

The current study identified significantly larger areas at the lesion epicenter that were immunopositive for CaMK-IIα, NF-200, and PECAM-1 in the transplantation group relative to the vehicle control group, suggesting that the grafted hiPSC-NS/PCs increased axonal sparing/regrowth as well as angiogenesis after SCI. The details of mammalian central pattern generator (CPG), which is responsible for coordinated locomotor function, remain elusive although the spinal locomotor CPG is better characterized in rodents after functional recovery after SCI [46]. Furthermore, descending fibers from the motor cortex are more crucial for motor functions, including dexterous hand movements, in primates than in rodents [47]. The increased sparing/regrowth of the descending axons induced by hiPSC-NS/PCs may contribute to functional recovery after SCI. Furthermore, NT3, NT4, and CNTF encourage axonal sparing/regrowth after SCI [29], [30], [31], [48], [49] and VEGF enhances angiogenesis at the lesion site by promoting cell survival pathways and inhibiting cell death [50], [51]. Importantly, cultured hiPSC-NS/PCs expressed significantly higher levels of NT3/4, CNTF and VEGF mRNA than hDFs, from which the hiPSCs were originally derived.

Transplanted NS/PCs not only promote replacement but also exert immunomodulatory and neuroprotective effects to prevent tissue damage [52], [53]. Furthermore, undifferentiated NS/PCs persisting in the injured spinal cord can secrete trophic factors capable of protecting endogenous neural cells from cell death, preventing glial scar formation, and increasing endogenous re-myelination along with immunomodulatory molecules [52], [53], [54], [55]. We performed Iba1 staining to assess microglial activation (Fig. 6A-1,A-2) and GFAP staining to assess glial scar formation (Fig. 6B). However, the size of the Iba1-positive area was not significantly different between control and transplantation groups, and there was no evidence of immunomodulation by hiPSC-NS/PCs at this time point (12 weeks after transplantation).

Previous studies demonstrated that the transplantation of oligodendrocyte progenitor cells derived from human ESCs can increase re-myelination after SCI in rodents [56]. Our recent study also revealed that remyelination was significant in terms of functional recovery after NS/PC transplantation into SCI mice [57]. In the present study, we confirmed that grafted hiPSC-NS/PCs survived in the host spinal cord and differentiated into Olig1-positive oligodendrocyte progenitor cells. However, the hiPSC-NS/PCs did not differentiate into mature myelin basic protein (MBP)-positive oligodendrocytes during the course of the experiment, perhaps because the period of observation was too short. By contrast, MRI and LFB and EC staining revealed that cell transplantation increased the size of the residual, or spared, myelination areas at the lesion site. These findings suggest that the grafted hiPSC-NS/PCs contributed to the preservation of myelin sheaths rather than to re-myelination after SCI. It is possible that mature oligodendrocytes appear at a later time-point, as oligodendrocyte progenitor cells were present in the grafted cells.

In conclusion, NS/PCs derived from the pre-evaluated safe human iPSC clone 201B7 survived and differentiated into three neural lineage cells within the injured spinal cord of adult common marmosets, with no evidence of tumor formation. The hiPSC-NS/PCs also contributed to significant functional recovery in this non-human primate model of SCI. Although hiPSCs hold great promise as a cell source for the study and treatment of human diseases and disorders [58], [59], it is critical to carefully assess safety concerns prior to their use in the clinic [60]. In particular, hiPSCs generated using retroviruses show integration of the virus DNA into host chromosomes [61], which may lead to unpredictable genetic dysfunction. Therefore, further study using integration-free hiPSC [62], [63], [64], [65], [66], [67], [68], [69], [70], [71]-derived NS/PCs is required to validate the safety of such cells for stem cell interventions following SCI.
